# Risk and protective factors for the development of gambling-related harms and problems among Australian sexual minority men

**DOI:** 10.1186/s40359-021-00597-4

**Published:** 2021-06-29

**Authors:** Rachel Bush, Alex M. T. Russell, Petra K. Staiger, Andrea Waling, Nicki A. Dowling

**Affiliations:** 1 School of Psychology, Deakin University, 221 Burwood Highway, BurwoodGeelong, 3125 Australia; 2 Experimental Gambling Research Laboratory, School of Health, Medical and Applied Sciences, CQUniversity, Level 6, 400 Kent St, Sydney, NSW 2000 Australia; 3 Centre for Drug Use, Addictive and Anti-Social Behaviour Research (CEDAAR), Deakin University, Geelong, Australia; 4 Australian Research Centre in Sex, Health and Society, NR6, La Trobe University, Bundoora, 3086 Australia; 5 Melbourne Graduate School of Education, University of Melbourne, Parkville, Australia

**Keywords:** Problem gambling, Gambling-related harms, Risk factors, Protective factors, Sexual minority men, Men

## Abstract

**Background:**

Sexual minority men (SMM) often experience stressful social environments dominated by stigma and discrimination. SMM are typically more likely than heterosexual men to engage in certain risky behaviours such as problem gambling. This study aimed to compare gambling behaviour among SMM and examine potential risk factors (erroneous gambling cognitions, gambling outcome expectancies, hazardous alcohol use, impulsivity, and psychological distress; as well as perceived stigma and discrimination for the SMM participants) and potential protective factors (resilience, social support, and community connectedness) for problem gambling severity and gambling-related harms among SMM living in Australia.

**Methods:**

An online survey, with an over-representation of SMM participants and problem, moderate-risk, and low-risk gamblers, was completed by 101 SMM (mean age = 28.5) and 207 heterosexual men (mean age = 26.4).

**Results:**

SMM were found to have significantly lower levels of problem gambling severity compared with heterosexual men, and report significantly lower gambling participation, frequencies and expenditure on any gambling activity, casino table games, horse racing/greyhound betting, sports betting, and keno. However, in the SMM group, 38.3% were classified in the problem gambling category of the Problem Gambling Severity Index and 27.6% were classified in the moderate-risk gambling category. There were no significant differences between groups in gambling-related harms. Multiple regression analyses revealed that problem gambling severity and related harms were independently predicted by higher levels of impulsivity and erroneous gambling cognitions for both groups.

**Conclusions:**

Lower frequency of gambling behaviours among SMM and similar risk factors predicting problem gambling severity/harms for both groups suggests that problem gambling is not pronounced among SMM. This study adds new evidence to the gambling literature which can be used as comparative benchmarks for future research.

**Supplementary Information:**

The online version contains supplementary material available at 10.1186/s40359-021-00597-4.

## Background

The Diagnostic and Statistical Manual of Mental Disorders, 5th edition [DSM-5]; [1] defines gambling disorder as “persistent and recurrent problematic gambling behaviour in a 12-month period which leads to substantial impairment or distress” (p. 585). In contrast, many jurisdictions, including Australia, have adopted a public health framework in which gambling problems are viewed across a risk continuum, from no-risk gambling characterised by an absence of adverse gambling-related harm to problem gambling characterised by severe harms [2]. The Australian national definition of problem gambling, which is “difficulties in limiting money and/or time spent on gambling which leads to adverse consequences for the gambler, others, or for the community”, reflects this continuum of risk [2].

Problem gambling is a serious world-wide public health concern, with the average international standardised prevalence rate estimated to be 2.3% [3]. In Australia, 0.4–0.6% of the population engage in problem gambling, 1.9–3.7% in moderate-risk gambling, and 3.0–7.7% in low-risk gambling [4, 5]. The harms experienced by gamblers and their families include impaired relationships, emotional distress, and financial problems [6, 7], and psychiatric conditions such as depression, anxiety and substance use are highly comorbid with problem gambling [8, 9]. Indeed, recent evidence suggests that the burden of harm resulting from gambling-related problems is of a level approximately two-thirds that of alcohol abuse/dependence and major depressive disorder [10]. It is therefore critical that we identify populations most at risk and understand the factors that might increase or decrease the risk of problem gambling behaviour.

A substantial amount of research has examined psychosocial correlates of problem gambling, with less research exploring the psychosocial correlates of gambling-related harms. Cross-sectional and longitudinal research suggests that key risk factors for the development of problem gambling include male gender, impulsivity, hazardous alcohol and/or drug use, depression, anxiety, erroneous gambling cognitions, and gambling outcome expectancies [8, 9, 11–19]. However, many people exposed to these risk factors may never develop gambling problems which suggests that there are factors that have a protective role [20]. Although the focus of less research, key protective factors identified in predominantly cross-sectional research appear to include social support, social bonding (community connectedness), and resilience [21–29].

Male gender has been consistently associated with the development of problem gambling (see review [15]). Accordingly, several studies have been conducted comparing male gamblers to female gamblers (e.g. [23, 30–33]). However, sexual minority men (SMM), referring to men whose sexual “identity, orientation or practices differ from the majority of the surrounding society” [34], have seldom been the focus of gambling research. Yet, research suggests that SMM have a greater risk of engaging in high-risk behaviours, such as hazardous alcohol and drug use [35, 36]. Minority stress theory [37] is commonly used in research to explain these disparities. This theory posits that the unique and socially-based stress that is experienced by sexual minority populations due to living in a stressful social environment created by stigma, discrimination, and prejudice [37–39] places them at a greater risk of poorer health outcomes as these stressors are experienced in addition to those experienced by the general population and require an extra level of adaptive effort [37].

SMM may therefore have a particularly high risk of developing gambling problems. That is, SMM may have a heightened experience of the risk factors for problem gambling that have been observed in heterosexual populations, but may be at even greater risk than heterosexual men due to exposure to a set of additional risk factors resulting from their minority status. Only a limited number of studies, however, have examined differences in gambling behaviour and problem gambling between SMM and heterosexual men. The first study to examine these differences used data collected between 1938 to 1963 from the Kinsey Institute for Sex Research [40]. Given the changing societal attitudes towards people in the sexual and gender diverse communities, as well as the increased accessibility of gambling, however, it is important to examine the findings from contemporary studies. A recent study by Richard et al. [41] compared differences in past-year gambling behaviour (participation and frequency) and problem gambling between 137 gay male, 65 bisexual male and 10,305 heterosexual male American student-athletes. Although no significant differences were found between the participant groups on past year gambling frequency, a significantly greater proportion of gay male participants participated in internet casino gambling (16%) and horse/greyhound race betting (14%) compared with bisexual male participants (8% and 8% respectively) and heterosexual male participants (6% and 6% respectively). Moreover, gay male and bisexual male participants were found to endorse a significantly higher number of DSM-5 gambling disorder criteria than heterosexual male participants.

Research examining the correlates of problem gambling among SMM is also limited, with only one cross-sectional study comparing heterosexual men and SMM conducted to date [42]. An American study by Grant and Potenza [42] examined the clinical correlates of problem gambling in a sample of 105 male treatment-seeking gamblers, 79.0% of whom were heterosexual, 14.3% who identified as gay, and 6.7% who identified as bisexual. Compared with heterosexual male participants, gay and bisexual male gamblers were significantly more likely to suffer from current impulse control disorders (68.2% versus 34.9%) and substance use disorders (59.1% versus 31.3%), likely due to living in a stressful social environment created by discriminatory and prejudicial attitudes [37].

Taken together, these findings suggest that SMM are potentially more at-risk of increased gambling participation and problem gambling than heterosexual men and are more likely to report comorbid impulse control and substance use disorders. However, additional research from other jurisdictions and populations is required to confirm these findings and to understand what other factors, such as depression, anxiety, erroneous gambling cognitions, and gambling outcome expectancies, might predict problem gambling among SMM. Moreover, factors relating to minority stress, such as perceived stigma relating to one’s sexual and/or gender identity and discrimination, have also not been examined as risk factors. Finally, factors which have been found to significantly protect SMM from the development of other high-risk behaviours, such as resilience, social support and community connectedness, have also not been examined in relation to problem gambling [43–45]. It is evident that further research is needed to examine the risk and protective factors for the development of problem gambling and gambling-related harms in SMM populations.

### Study aims and hypotheses

This study therefore aimed to examine gambling behaviour (participation, frequency, expenditure), problem gambling, and gambling-related harm among SMM, as well as the risk factors and protective factors that uniquely predict problem gambling and gambling-related harms among SMM compared with heterosexual men. It was hypothesised that: (1) SMM would report significantly higher (a) gambling participation, frequency and expenditure, (b) problem gambling severity, and (c) gambling-related harms than heterosexual men; (2) risk factors (erroneous gambling cognitions, gambling outcome expectancies, hazardous alcohol use, impulsivity, and psychological distress; as well as perceived stigma and discrimination for the SMM participants) would predict higher problem gambling severity and gambling-related harms in both SMM and heterosexual men; (3) the relationship between these risk factors and problem gambling severity/gambling-related harms would be more pronounced among SMM than heterosexual men; (4) protective factors (resilience, social support, and community connectedness) would predict lower problem gambling severity and gambling-related harms in both SMM and heterosexual men; and (5) the relationship between these protective factors and problem gambling severity/gambling-related harms would be less pronounced among SMM than heterosexual men.

## Methods

### Recruitment and sampling

The data examined in this study was derived from a larger study examining risk and protective factors for problem gambling and related harms among sexual and gender minority communities. The convenience sample consisted of heterosexual, lesbian, gay, bisexual, transgender, intersex, queer, and other sexually and gender diverse (LGBTIQ +) people living in Australia and aged 18 years or older. The study was approved by the relevant University Human Research Ethics Committee (reference number: 2017-077), and by a prominent LGBTIQ + community health organisation, the Thorne Harbour Health Community Research Endorsement Panel (reference number: THH/CREP/19/002). Recruitment commenced February 28, 2019 and ended November 30, 2019. Participants were recruited from three sources, with an over-sampling of LGBTIQ + people and people with gambling problems: (1) state- and nation-wide general and sexual and gender minority community and social networks; (2) public common areas; and (3) prominent sexual and gender minority organisations. All study advertisements included a link for an online survey hosted on the Qualtrics survey platform. Participants went into a draw to win one of six AUD$50 retail vouchers.

Eight hundred and eighteen eligible participants who lived in Australia and were aged 18 years and older started the survey. Of these participants, 531 identified as male (sexual minority and heterosexual). Of these male participants, 311 completed all measures, however, three participants were removed as two revealed significant amounts of missing data and were excluded from analysis as per the protocol recommended by Jakobsen and colleagues [46] and one participant displayed evidence of straight-lining (i.e. selecting the same response through all scales). The resulting sample comprised 308 male participants (207 heterosexual men, 101 SMM).

### Measures

#### Demographic characteristics

Standard demographic questions were included, including age, country of birth, indigeneity, residential location, education, and occupational status.

#### Gender identity and sexuality

*Gender identity* Participants were asked, “Which of the following best describes your gender identity? Please select all that apply” with response options: male, female, trans female/trans woman, trans male/trans man, non-binary/gender fluid, agender, and other (please describe). Participants were also asked, “What gender were you assigned at birth (i.e. what was specified on your original birth certificate)?” with male and female as response options. Intersex status was collected by asking “Were you born with a variation of sex characteristics? (this is sometimes called 'intersex')” with response options of yes, no, and prefer not to answer. All participants who identified as male and were assigned male at birth were retained for analysis in the current paper.

*Sexuality* Participants answered questions about their sexual identity, attraction, and behaviour. To measure identity, participants were asked, “Do you consider yourself to be” with the following response options: lesbian, gay, bisexual, queer, pansexual, asexual, heterosexual/straight, other (please specify). For analyses in the current paper, responses to this question were used to classify participants with participants selecting heterosexual/straight being classified into the heterosexual group and participants endorsing any other option being classified into SMM group. To measure attraction, participants were asked, “Which of the following best describes who you are sexually attracted to? (Please select all that apply)” with the following response options: women, men, non-binary/gender fluid individuals, different identity (please specify). To measure behaviour, participants were asked about their relationship status: “Are you currently in a relationship?” with response options yes with one person, yes with more than one person, no. Those in a relationship with one person were asked “Are you in a relationship with:” and were provided with the following response options: a woman, a man, a trans woman, a trans man, non-binary individual, other (please specify). Participants who were in a relationship with more than one person were asked to type in the gender identities and sexual orientations of their partners.

#### Gambling measures

*Gambling participation, frequency and expenditure* Questions about participation and frequency of gambling were modelled from the Victorian Prevalence Survey 2014 [47] and questions on expenditure where modelled from the Social and Economic Impact Study of Gambling in Tasmania [48]. The survey included a list of 10 types of gambling and participants answered yes or no to which they had spent money on in the past 12 months: informal private games for money; pokies or electronic gaming machines; casino table games; horse, harness, or greyhound racing; sports; events; keno; lotteries, powerball or the pools; instant scratch tickets; and bingo. For each gambling activity, participants then answered questions about modality, gambling frequency, and gambling expenditure in the previous 12 months.

*Problem gambling severity* Past-year problem gambling severity was measured using the Problem Gambling Severity Index (PGSI) [49], which includes 9 items answered on a 4-point Likert scale ranging from 0 (never) to 3 (almost always). Scores range from 0 to 27, which can be classified into four categories of symptom severity: non-problem gambling (scores of 0), low-risk gambling (scores of 1–2), moderate-risk gambling (scores of 3–7), and problem gambling (scores of 8 or more). Good internal consistency has been demonstrated for the PGSI [49, 50]. Cronbach’s alpha was 0.94 and the Omega coefficient was also 0.94 for the current sample which indicated excellent internal reliability.

*Gambling-related harms* Past-year gambling-related harms were measured using the Short Gambling Harms Screen (SGHS) [51], a 10-item measure which asks yes/no questions about gambling harms. Scores greater than zero (i.e. one or more items endorsed) indicate a presence of gambling-related harms, with higher scores indicating more harm. The SGHS has demonstrated very strong reliability [51]. Cronbach’s alpha was 0.90 and the Omega coefficient was 0.89 for the current sample.

#### Psychosocial factors

*Erroneous gambling cognitions* The 23-item Gambling Related Cognition Scale (GRCS) [52] was used to measure erroneous gambling cognitions. Items are scored on a 7-point Likert scale ranging from 1 (strongly disagree) to 7 (strongly agree). The scale produces a total score and five subscales: Gambling expectancies (for example, “Gambling makes me happier”); Illusion of control (for example, “Praying helps me win”); Predictive control (for example, “When I have a win once, I will definitely win again”); Inability to stop gambling (for example, “I will never be able to stop gambling”); and Interpretive bias (for example, “Relating my losses to probability makes me continue gambling”). Higher scores indicate more erroneous gambling cognitions. Good psychometric properties have demonstrated that this is a useful tool for identifying erroneous gambling cognitions among non-clinical gamblers [52]. Cronbach’s alpha for the total score and the five subscales for the current sample were 0.92, 0.69, 0.74, 0.75, 0.90, and 0.76, respectively. The Omega coefficient for the total score and the five subscales for the current sample were 0.94, 0.70, 0.75, 0.75, 0.90, and 0.76, respectively.

*Gambling outcome expectancies* Gambling outcome expectancies were measured using the Gambling Expectancy Questionnaire (GEQ) [53], which consists of 23 items asking participants the likelihood of each outcome when they are gambling. Items are answered on a 7-point Likert-type scale ranging from 1 (no chance) to 7 (certain to happen). The scale consists of three subscales measuring positive outcome expectancies (enjoyment/arousal, self-enhancement, and money) and two subscales measuring negative outcome expectancies (overinvolvement, emotional impact). Higher scores indicate a higher level of expectancies about gambling. This scale has been found to have strong validity and adequate to good internal reliability [53]. For the current study, Cronbach’s alphas were 0.86, 0.79 and 0.83, respectively, for the positive outcome expectancy subscales (enjoyment/arousal, self-enhancement, and money), respectively; and 0.90 and 0.93 for the negative outcome expectancy subscales (overinvolvement, emotional impact), respectively. The Omega coefficients were 0.87, 0.79 and 0.86 for the positive outcome expectancy subscales, and 0.90 and.93 for the negative outcome expectancy subscales.

*Hazardous alcohol use* The Alcohol Use Disorders Identification Test—Consumption (AUDIT-C) [54], which is a shortened 3-item version of the AUDIT, was used to measure hazardous alcohol use. Items are answered on differing 4-point Likert scales with higher scores indicating a greater severity of alcohol use. The AUDIT-C has been found to be a valid and reliable measure for assessing problem drinking [55]. Cronbach’s alpha was 0.75 and the Omega coefficient was 0.78 for the current sample.

*Impulsivity* Impulsivity was measured using the (Negative) Urgency subscale of the UPPS-P Impulsive Behaviour Scale (UPPS-P) [56], which consists of 12 items answered on a 4-point Likert scale ranging from 1 (strongly agree) to 4 (disagree strongly). High scores indicate difficulty with resisting temptations. Evidence of the validity of this scale has been found in various populations [57, 58]. Cronbach’s alpha was 0.94 and the Omega coefficient was also 0.94 for the current sample.

*Psychological distress* Psychological distress was measured using the 6-item Kessler Psychological Distress Scale (K6) [59], with higher scores indicating more psychological distress. Items are answered on a 5-point Likert scale ranging from 1 (all of the time) to 5 (none of the time) and reverse coded for analysis. The reliability and validity of this scale have been demonstrated in various populations, such as adolescents, and adults with co-occurring substance use disorders [60, 61]. Cronbach’s alpha was 0.91 and the Omega coefficient was also 0.91 for the current sample.

*Perceived stigma* Perceived stigma was measured using the Perceptions of Local Stigma Scale (PLS) [62], which includes 7 items answered on a 5-point Likert scale ranging from 1 (strongly disagree) to 5 (strongly agree), with higher scores indicating a greater level of perceived stigma. This measure was modified to refer to “where I live” instead of “the Sacramento area”. Good reliability has previously been found for this scale [62]. In the current study, reliability was also good as indicated by a Cronbach’s alpha of 0.88 and an Omega coefficient of 0.87.

*Experiences of discrimination* Discrimination experiences were measured using a dichotomous item [63], which was adapted for the current study. The original question asks, “Sometimes people feel they are discriminated against or treated badly by other people. In the past 12 months, have you felt discriminated against because someone thought you were gay, lesbian, or bisexual?” However, this study asked, “Sometimes people feel they are discriminated against or treated badly by other people. In the past 12 months, have you felt discriminated against because of your sexual and/or gender identity?” The question was modified as the survey originally collected data from all sexual and gender minorities. It was answered using a 5-point Likert scale ranging from 0 (not at all) to 4 (very much).

*Resilience* Resilience was measured using the Brief Resilience Scale (BRS) [64], which is a 6-item scale assessing the ability to recover from stress with items answered on a 5-point Likert scale ranging from 1 (strongly disagree) to 5 (strongly agree). Higher scores indicate a greater level of resilience. Strong internal reliability has been demonstrated for the BRS [64]. Cronbach’s alpha was 0.85 and the Omega coefficient was also 0.85 for the current sample.

*Social support* Social support was measured using the Medical Outcomes Study Social Support Survey (MOS-SS) [65], which contains 19 items asking how often each type of support was available if needed. Items were answered on a 5-point Likert scale ranging from 1 (none of the time) to 5 (all of the time), with higher scores indicating more social support. Questions relate to emotional/informational support, tangible support, affectionate support, and positive social interaction. Excellent internal reliability has been demonstrated for the total score [65]. Cronbach’s alpha was 0.97 for the current sample’s total score and 0.95, 0.89, 0.92 and 0.92, respectively, for the subscales. The Omega coefficient for the total score and subscales was 0.98, 0.94, 0.89, 0.92 and 0.92, respectively.

*Community connectedness* Community connectedness was measured using an adapted version of the Connectedness to the LGBT Community Scale [66]. In the current study, this scale referred to the LGBTIQ + community in general rather than the LGBTI community in New York. A modified version that was used in the Rainbow Women’s Help-Seeking Study was also included to measure connectedness to the mainstream community [67]. Both scales consist of 7 items answered on a 4-point Likert scale ranging from 1 (strongly disagree) to 4 (strongly agree), with higher scores indicating a greater level of connectedness to the LGBTIQ + and/or mainstream community. Good internal reliability has been demonstrated for the LGBTIQ + and mainstream versions in an Australian sample of sexual minority women [67]. In the current sample, Cronbach’s alpha scores were 0.89 and 0.88 and the Omega coefficient was 0.89 and 0.89 for the LGBTIQ + community connectedness and the mainstream community connectedness scales, respectively.

### Data analysis

The percentage of missing values for the entire sample ranged from 0.3% for some of the PGSI, SGHS, GRCS, GEQ, UPPS-P, K6, MOS-SS and mainstream community connectedness items, to 1.3% for one MOS-SS item. Among the SMM participants, the percentage of missing values for the LGBTIQ community connectedness scale ranged from 1.0% for three items to 2.9% for one item. Data are primarily missing due to item nonresponse and given that it was found to be missing completely at random, multiple imputation was conducted for all analysis variables [68]. If more than 40% of items were missing in a scale, the participant was excluded from analysis [46]. Missing data was not imputed for perceived discrimination as this measure contained one item. Using SPSS’s ‘multiple imputation’ command, 10 imputed datasets were generated. Analyses run on each dataset were pooled according to Rubin’s rules [69]. The imputed values compare reasonably to observed values so imputed results are presented for the bivariate regression analyses and the multivariate regression analyses. The PGSI was log (+ 1) transformed to reduce skewness. All statistical tests were conducted with an alpha level of 0.05.

Participant characteristics and gambling behaviour (hypothesis 1) were compared between the heterosexual group and the SMM group using Chi-square or Fisher’s exact tests for categorical variables, and independent samples *t*-tests or Mann–Whitney tests for numerical variables. Separate bivariate regression analyses were conducted for the heterosexual group and the SMM group to determine which psychosocial factors (including the planned covariates employed in subsequent multivariate analyses) were significantly associated with PGSI scores and SGHS scores. Using the significant factors, four multiple regression analyses were performed (two for the heterosexual group and two for the SMM group) to determine which of these factors predicted higher PGSI scores and higher SGHS scores when controlling for age, relationship status, tertiary education, and number of gambling activities (hypotheses 2 and 4). Due to issues of multicollinearity (tolerance < 0.4), these multiple regression analyses were performed again after removing the GEQ subscales and K6 psychological distress, and using only the total scores for GRCS erroneous gambling cognitions and MOS-SS social support. Finally, a series of moderation analyses were performed using multivariate regression to examine the degree to which sexual minority status moderated the relationship between each psychosocial factor and PGSI scores/SGHS scores (hypotheses 3 and 5).

## Results

### Participant characteristics

Table 1 displays the demographic characteristics of the heterosexual and SMM participants. The sexual orientations of the SMM participants were varied, with the largest proportions of participants identifying as gay (51%) and bisexual (35%), with an additional 10% identifying as pansexual, and only one participant identifying as queer and one participant identifying as asexual. The three SMM participants who selected “other” reported a sexual orientation that was not included in the list or multiple sexual orientations: (1) non-classified; (2) gay and queer; and (3) “I have sex with people I find attractive. Gender is like last [sic] question on my mind, but I find femininity the most attractive.” Three participants were identified as having intersex variations. A significantly greater proportion of SMM participants had completed a university degree compared with the heterosexual group, however, they were also significantly more likely to be unemployed while a significantly greater proportion of heterosexual participants were employed full-time.

**Table 1:** Comparisons in demographic characteristics between heterosexual male and sexual minority male participants

Characteristics	Het. Men (*n* = 207)	SMM (*n* = 101)	Inferential statistics	Effect size
Age, *M* (*SD*), years	26.4 (11.2)	28.5 (11.5)	*t* (306) = − .1.60, *p* = .112	*d* = .19
Sexual orientation, *n* (%)				
Gay	0	51 (50.5)		
Bisexual	0	35 (34.7)		
Queer	0	1 (1.0)		
Pansexual	0	10 (9.9)		
Asexual	0	1 (1.0)		
Heterosexual	207 (100.0)	0		
Other	0	3 (3.0)		
Indigeneity (yes), *n* (%)	6 (2.9)	1 (1.0)	χ^2^ (1, n = 308) = .42, *p* = .517	Φ = − .06
Live alone (yes), *n* (%)	21 (10.1)	18 (17.8)	χ^2^ (1, n = 308) = 2.96, *p* = .086	Φ = .11
Relationship (yes), *n* (%)	112 (54.1)	47 (46.5)	χ^2^ (1, n = 308) = 1.27, *p* = .260	Φ = − .07
Currently live, *n* (%)			χ^2^ (2, n = 307) = .29, *p* = .860	Φ = .03
Urban area	145 (70.4)	72 (71.3)		
Regional centre^a^	28 (13.6)	15 (14.9)		
Rural area^b^	33 (16.0)	14 (13.9)		
Education, *n* (%)			**χ**^**2**^** (2, n = 308) = 10.67, *p* = .005**	**Φ = .19**
Primary/secondary school	128 (61.8)	53 (52.5)		
Certificate/diploma	43 (20.8)	14 (13.9)		
University degree	36 (17.4)	34 (33.7)*		
Occupational status, *n* (%)			**Fisher’s = 14.82, *p* = .003**^**†**^	**Φ = .22**
Part-time	72 (35.0)	35 (34.7)		
Full-time	100 (48.5)*	32 (31.7)		
Unpaid work (including home duties)	2 (1.0)	2 (2.0)		
Unemployed, seeking work	22 (10.6)	26 (25.7)*		
None of these	10 (4.9)	6 (5.9)		

Rows in bold indicate significant differences between groups. *d* = Cohen’s *d.* Φ = Phi. SMM = sexual minority men

*Indicates that the proportion of respondents in that category from that group (either heterosexual male or sexual minority male participants) is significantly higher than the proportion of respondents from the other group.

^†^Fisher’s Exact test two-sided

^a^Population 50,000 or more

^b^Population less than 50,000

Compared with the SMM group, the heterosexual group reported significantly higher levels of gambling-related expectancies, interpretive bias, enjoyment/arousal outcome expectancies, self-enhancement outcome expectancies, over-involvement outcome expectancies, resilience, emotional/informational support, tangible support, affectionate support, and positive social interaction. Compared with the heterosexual group, the SMM group reported significantly higher levels of psychological distress. No significant group differences were found for illusion of control, predictive control, inability to stop gambling, money outcome expectancies, emotional impact expectancies, hazardous alcohol use, impulsivity, or mainstream community connectedness (see Additional file 1: Appendix A in the supplementary materials for statistical group comparisons in each of the potential risk factors and potential protective factors).

### Gambling behaviour

A significantly greater proportion of heterosexual males reported participating in any gambling activity, specifically casino table games, horse/greyhound race betting, sports betting, and keno compared with the SMM participants (see Table 2) over the last 12 months. Furthermore, heterosexual men reported engaging in gambling activities significantly more frequently overall and engaging in casino table games, horse/greyhound race betting, sports betting, and keno significantly more frequently than SMM (see Table 2) and spent significantly more money on average overall and on these gambling activities during each gambling session (see Table 2).

**Table 2:** Past 12-month participation in gambling activities, gambling frequency^a^, and expenditure^b^

Gambling participation	Het. men *n* (%)	SMM *n* (%)	Inferential statistics	Φ
Informal private games	81 (39.1)	31 (30.7)	χ^2^ (1, n = 308) = 1.74, *p* = .187	− .08
Pokies/electronic gaming machines	136 (65.7)	63 (62.4)	χ^2^ (1, n = 308) = .20, *p* = .656	− .03
**Casino table games**	**133 (64.3)**	**39 (38.6)**	**χ**^**2**^** (1, n = 308) = 17.07, *p* < .001**	− .**24**
**Horse racing/greyhounds**	**153 (73.9)**	**47 (46.5)**	**χ**^**2**^** (1, n = 308) = 21.16, *p* < .001**	− .**27**
**Sports**	**151 (72.9)**	**37 (36.6)**	**χ**^**2**^** (1, n = 308) = 36.13, *p* < .001**	− .**35**
Novelty bets^c^	36 (17.4)	16 (15.8)	χ^2^ (1, n = 308) = .03, *p* = .858	− .02
**Keno**	**77 (37.2)**	**21 (20.8)**	**χ**^**2**^** (1, n = 308) = 7.68, *p* = .006**	− .**17**
Lotto/Powerball	103 (49.8)	48 (47.5)	χ^2^ (1, n = 308) = .06, *p* = .805	− .02
Instant scratch tickets	74 (35.7)	44 (43.6)	χ^2^ (1, n = 308) = 1.44, *p* = .230	.08
Bingo	6 (2.9)	6 (5.9)	χ^2^ (1, n = 308) = .96, *p* = .326	.07
**Any gambling participation (ref = yes)**	**204 (98.6)**	**94 (93.1)**	**χ**^**2**^** (1, n = 308) = 4.87, *p* = .027**	− .**15**

	Het. men		SMM			
Gambling frequency^a^	Median	IQR	Median	IQR	Inferential statistics	*r*
Informal private games	0.0	5.0	0.0	0.0	*U* = 8435.00, *z* = − .1.56, *p* = .118	− .09
Pokies/electronic gaming machines	4.0	36.0	4.0	39.0	*U* = 9185.50, *z* = − .91, *p* = .365	− .05
** Casino table games**	**2.0**	**12.0**	**0.0**	**3.3**	***U* = 7160.00, *z* = **− .**4.38, *p* < .001**	− .**25**
** Horse racing/greyhounds**	**52.0**	**208.0**	**0.0**	**80.0**	***U* = 6330.00, *z* = **− .**5.27, *p* < .001**	− .**31**
** Sports**	**36.0**	**117.0**	**0.0**	**10.5**	***U* = 5395.00, *z* = **− .**6.41, *p* < .001**	− .**37**
Novelty bets^c^	0.0	0.0	0.0	0.0	*U* = 9494.50, *z* = − .71, *p* = .479	− .04
** Keno**	**0.0**	**3.3**	**0.0**	**0.0**	***U* = 8362.50, *z* = **− .**2.85, *p* = .004**	− .**16**
Lotto/Powerball	0.0	5.3	0.0	12.0	*U* = 9975.50, *z* = .04, *p* = .968	.00
Instant scratch tickets	0.0	2.0	0.0	4.0	*U* = 10,020.00, *z* = 1.15, *p* = .249	.07
Bingo	0.0	0.0	0.0	0.0	*U* = 10,779.50, *z* = 1.33, *p* = .185	.08
** Total gambling frequency**	**244.0**	**427.0**	**76.0**	**197.5**	***U* = 6649.00, *z* = **− .**5.19, *p* < .001**	− .**30**

Gambling expenditure (AUD$)^b^						
Informal private games	0.0	20.0	0.0	0.0	*U* = 8022.00, *z* = − .1.31, *p* = .189	− .08
Pokies/electronic gaming machines	10.0	70.0	20.0	100.0	*U* = 8734.50, *z* = − .24, *p* = .813	− .01
** Casino table games**	**35.0**	**200.0**	**0.0**	**50.0**	***U* = 6550.00, *z* = **− .**4.16, *p* < .001**	− .**25**
** Horse racing/greyhounds**	**20.0**	**100.0**	**0.0**	**25.0**	***U* = 5808.50, *z* = **− .**4.42, *p* < .001**	− .**27**
** Sports**	**10.0**	**50.0**	**0.0**	**20.0**	***U* = 5579.00, *z* = **− .**5.05, *p* < .001**	− .**30**
Novelty bets	0.0	0.0	0.0	0.0	*U* = 9373.00, *z* = − .79, *p* = .427	− .05
** Keno**	**0.0**	**10.0**	**0.0**	**0.0**	***U* = 8252.50, *z* = **− .**2.70, *p* = .007**	− .**16**
Lotto/Powerball	0.0	25.0	0.0	20.0	*U* = 9398.00, *z* = − .25, *p* = .804	− .01
Instant scratch tickets	0.0	5.0	0.0	6.3	*U* = 9884.50, *z* = 1.28, *p* = .200	.08
Bingo	0.0	0.0	0.0	0.0	*U* = 10,775.50, *z* = 1.58, *p* = .113	.09
**Total gambling expenditure**	**215.0**	**575.0**	**80.0**	**260.0**	***U* = 7977.00, *z* = **− .**3.38, *p* = .001**	− .**19**

Rows in bold indicate significant differences between groups. IQR = interquartile range. SMM = sexual minority men. Φ = Phi

^a^Average number of times participants gambled in the previous 12 months

^b^Amount of money (AUD$) spent on average during each gambling session in the previous 12 months. Expenditure represents the difference between what the participant took with them (including any additional money withdrawn or borrowed during the period of betting) and what they had left when they finished. ^c^Such as, election results, current affairs and TV shows

### Problem gambling and related harms

Comparison of mean PGSI total scores revealed that heterosexual men reported significantly higher problem gambling severity than SMM (see Table 3). There were no significant differences in PGSI risk categories between the participant groups. The largest proportions of participants in both groups were classified in the problem gambling and the moderate-risk gambling PGSI categories. There was no significant difference in the level of gambling-related harm (SGHS scores) between SMM and heterosexual men.

**Table 3:** Comparisons in problem gambling severity and gambling-related harm between heterosexual male and sexual minority male participants

Characteristics	Het. men	SMM	Inferential statistics	Effect size
**Problem gambling severity**^**a**^**, *M* (*SD*)**	1.8 (1.0)*	**1.5 (1.0)**	***t* (2302) = 2.00 *p* = .047**	***d* = **− .**3**
Gambling risk category^a^, *n* (%)			*χ2* (3, *n* = 304) = 4.10, *p* = .250	Φ = .12
Non-problem gambling	25 (12.3)	21 (21.0)		
Low-risk gambling	40 (19.6)	18 (18.0)		
Moderate-risk gambling	58 (28.4)	27 (27.0)		
Problem gambling	81 (39.7)	34 (34.0)		
Gambling-related harms^b^, *M* (*SD*)	3.8 (3.4)	3.4 (3.3)	*t* (300) = .85 *p* = .397	*d* = − .12
Gambling-related harms^b^ score ≥ 1, *n* (%)	151 (75.1)	72 (71.3)	*χ2* (1, *n* = 302) = .33*, p* = .564	Φ = − .04

Rows in bold indicate significant differences between groups. *d* = Cohen’s *d.* χ2 = Chi-square. Φ = Phi

^*^Indicates that the proportion of respondents in that category from that group (either heterosexual male or sexual minority male participants) is significantly higher than the proportion of respondents from the other group

^a^Problem Gambling Severity Index. Total score range = 0–27; Non-problem gambling = 0; Low-risk gambling = 1–2; Moderate-risk gambling = 3–7; Problem gambling = 8 or more

^b^Short Gambling Harms Screen. Score range = 0–10

### Psychosocial factors associated with problem gambling severity and related harms

Table 4 displays the bivariate and multiple regression analyses predicting PGSI scores and SGHS scores for the heterosexual men and the SMM. Bivariate regression analyses in the heterosexual group revealed that age, being in a relationship, money expectancies, resilience, emotional informal support, tangible support, affectionate support and positive social interaction significantly negatively predicted PGSI scores; and that number of gambling activities, gambling-related expectancies, illusion of control, predictive control, inability to stop gambling, interpretive bias, self-enhancement expectancies, over-involvement expectancies, emotional impact expectancies, hazardous alcohol use, impulsivity and psychological distress significantly positively predicted PGSI scores. Money expectancies, resilience, emotional informational support, tangible support, affectionate support and positive social interaction significantly negatively predicted SGHS scores; and number of gambling activities, gambling-related expectancies, illusion of control, predictive control, inability to stop gambling, interpretive bias, over-involvement expectancies, emotional impact expectancies, hazardous alcohol use, impulsivity and psychological distress significantly positively predicted SGHS scores.

**Table 4:** Unstandardised betas (and 95% confidence intervals) from bivariate and multiple regression models predicting PGSI scores and SGHS scores with psychosocial correlates among heterosexual male participants and SMM participants

Model: PGSI scores	Het. Men		SMM	
	Bivariate	Multiple	Bivariate	Multiple
(Constant)		− .41 (−1.29, .48)		− .68 (−1.65, .30)
Age (in years)	− .01 (− .03, − .00)*	− .01 (− .02, .00)	.01 (− .01, .02)	−^a^
Relationship (ref=single)	− .36 (− .65, − .08)*	.06 (− .15, .27)	.26 (− .14, .67)	−^a^
Tertiary education (ref=no)	− .20 (− .49, .09)	−^a^	− .03 (− .44, .38)	−^a^
Number of gambling activities^b^	.15 (.09, .21)***	.06 (.02, .11)*	.24 (.16, .32)***	.16 (.08, .23)***
Erroneous gambling cognitions (GRCS total score)	−	.02 (.02, .03)***	−	.02 (.01, .02)***
Erroneous gambling expectancies (GRCS subscale)	.09 (.06, .12)***	−^c^	.10 (.07, .13)***	−^c^
Illusion of control (GRCS subscale)	.10 (.07, .12)***	−^c^	.06 (.02, .10)**	−^c^
Predictive control (GRCS subscale)	.06 (.04, .08)***	−^c^	.04 (.02, .07)**	−^c^
Inability to stop gambling (GRCS subscale)	.09 (.08, .10)***	−^c^	.10 (.08, .12)***	−^c^
Interpretive bias (GRCS subscale)	.11 (.08, .12)***	−^c^	.09 (.05, .12)***	−^c^
Enjoyment/arousal expectancies (GEQ subscale)	.00 (− .02, .02)	−^ac^	.04 (.01, .06)**	−^c^
Self-enhancement expectancies (GEQ subscale)	.03 (.00, .06)*	−^c^	.07 (.04, .11)***	−^c^
Money expectancies (GEQ subscale)	− .04 (− .08, − .01)*	−^c^	.05 (− .01, .10)	−^ac^
Over-involvement expectancies (GEQ subscale)	.09 (.08, .10)***	−^c^	.10 (.08, .12)***	−^c^
Emotional impact expectancies (GEQ subscale)	.14 (.12, .16)***	−^c^	.10 (.06, .13)***	−^c^
Hazardous alcohol use (AUDIT-C)	.09 (.04, .14)***	.01 (− .03, .05)	.04 (− .03, .12)	−^a^
Impulsivity (UPPS-P)	.73 (.58, .88)***	.34 (.18, .49)***	.57 (.31, .83)***	.35 (.13, .57)**
Psychological distress (K6)	.08 (.06, .10)***	−^c^	.03 (− .00, .07)	−^c^
Resilience (BRS)	− .34 (− .50, − .18)***	.05 (− .09, .18)	− .17 (− .41, .07)	−^a^
Social support (MOS-SS total score)	−	− .09 (− .20, .01)	−	−
Emotional informational support (MOS-SS subscale)	− .22 (− .35, − .09)***	−^c^	− .24 (− .41, − .06)*	−^c^
Tangible support (MOS-SS subscale)	− .16 (− .28, − .03)*	−^c^	− .13 (− .30, .04)	−^ac^
Affectionate support (MOS-SS subscale)	− .21 (− .33, − .10)***	−^c^	− .11 (− .27, .05)	−^ac^
Positive social interaction (MOS-SS subscale)	− .23 (− .36, − .09)***	−^c^	− .27 (− .44, − .09)**	−^c^
Mainstream community connectedness	− .02 (− .05, .01)	−^a^	− .05 (− .09, − .00)*	− .01 (− .05, .02)
LGBTIQ community connectedness	−^d^	−^d^	− .01 (− .05, .04)	−^a^
Perceived stigma	−^d^	−^d^	.05 (.01, .08)*	−^e^
Discrimination (ref=no)	−^d^	−^d^	− .06 (− .49, .36)	−^ae^
*R*^2^	−	.57	−	.50
*F*	−	31.55***	−	23.91***

Model: SGHS scores	Het. men		SMM	
	Bivariate	Multiple	Bivariate	Multiple
(Constant)		− 3.92 (− 6.88, − .97)*		− 1.15 (− 5.26, 2.97)
Age (in years)	− .03 (− .07, .01)	−^a^	.06 (.00, .11)*	.04 (− .00, .09)
Relationship (ref=single)	− .66 (− 1.58, .26)	−^a^	.76 (− .51, 2.04)	−^a^
Tertiary education (ref=no)	− .47(− 1.45, .45)	−^a^	.23 (− 1.04, 1.51)	−^a^
Number of gambling activities^b^	.43 (.24, .61)***	.13 (− .03, .29)	.49 (.22, .76)***	.26 (.00, .53)*
Erroneous gambling cognitions (GRCS total score)	−	.06 (.04, .07)***	−	.04 (.01, .07)**
Erroneous gambling expectancies (GRCS subscale)	.22 (.13, .32)***	−^c^	.21 (.10, .32)***	−^c^
Illusion of control (GRCS subscale)	.31 (.23, .39)***	−^c^	.20 (.07, .34)**	−^c^
Predictive control (GRCS subscale)	.16 (.10, .22)***	−^c^	.10 (.02, .19)*	−^c^
Inability to stop gambling (GRCS subscale)	.28 (.24, .32)***	−^c^	.27 (.20, .34)***	−^c^
Interpretive bias (GRCS subscale)	.27 (.20, .33)***	−^c^	.20 (.09, .30)***	−^c^
Enjoyment/arousal expectancies (GEQ subscale)	− .04 (− .11, .03)	−^ac^	.07 (− .01, .14)	−^c^
Self-enhancement expectancies (GEQ subscale)	.07 (− .02, .16)	−^ac^	.16 (.04, .28)**	−^c^
Money expectancies (GEQ subscale)	− .23 (− .34, − .11)***	−^c^	− .02 (− .18, .15)	−^ac^
Over-involvement expectancies (GEQ subscale)	.27 (.23, .32)***	−^c^	.27 (.20, .35)***	−^c^
Emotional impact expectancies (GEQ subscale)	.46 (.40, .51)***	−^c^	.36 (.26, .46)***	−^c^
Hazardous alcohol use (AUDIT-C)	.30 (.13, .46)**	.08 (− .05, .22)	.19 (− .04, .41)	−^a^
Impulsivity (UPPS-P)	2.27 (1.77, 2.77)***	1.27 (.70, 1.83)***	1.63 (.79, 2.47)***	.93 (.13, 1.73)*
Psychological distress (K6)	.25 (.18, .32)***	−^c^	.13 (.03, .23)*	−^c^
Resilience (BRS)	− 1.11 (− 1.62, − .59)***	.07 (− .41, .54)	− .60 (− 1.35, .15)	−^a^
Social support (MOS-SS total score)	−	− .08 (− .44, .28)	−	− .23 (− .79, .34)
Emotional informational support (MOS-SS subscale)	− .46 (− .88, − .04)*	−^c^	− .78 (− 1.34, − .22)**	−^c^
Tangible support (MOS-SS subscale)	− .48 (− .87, − .09)*	−^c^	− .45 (− .98, .08)	−^ac^
Affectionate support (MOS-SS subscale)	− .54 (− .92, − .17)**	−^c^	− .46 (− .95, .04)	−^ac^
Positive social interaction (MOS-SS subscale)	− .59 (− 1.02, − .16)**	−^c^	− .93 (− 1.48, − .38)***	−^c^
Mainstream community connectedness	− .04 (− .14, .07)	−^a^	− .18 (− .33, − .04)*	− .09 (− .22, .04)
LGBTIQ community connectedness	−^d^	−^d^	− .00 (− .13, .13)	−^a^
Perceived stigma	−^d^	−^d^	.20 (.10, .31)***	−^e^
Discrimination (ref=no)	−^d^	−^d^	− .01 (− 1.33, 1.31)	−^ae^
*R*^2^	−	.46	−	.34
*F*	−	26.61***	−	8.29***

*p < .05; **p < .01; ***p < .001. AUDIT-C = Alcohol Use Disorders Identification Test – Consumption. BRS = The Brief Resilience Scale. GEQ = Gambling Expectancy Questionnaire. GRCS = Gambling Related Cognitions Scale. K6 = The 6-item Kessler Psychological Distress Scale. MOS-SS = The Medical Outcomes Study Social Support Survey. UPPS-P = The (Negative) Urgency subscale of the UPPS-P Impulsive Behaviour Scale. PGSI = Problem Gambling Severity Index. SGHS = Short Gambling Harm Screen

Heterosexual group’s multiple regression model predicting PGSI scores included age, relationship status and number of gambling activities (as covariates), hazardous alcohol use, impulsivity, resilience, social support, and erroneous gambling cognitions

Heterosexual group’s multiple regression model predicting SGHS scores included number of gambling activities (as a covariate), hazardous alcohol use, impulsivity, resilience, social support, and erroneous gambling cognitions

SMM group’s multiple regression model predicting PGSI scores included number of gambling activities (as a covariate), impulsivity, mainstream community connectedness, and erroneous gambling cognitions

SMM group’s multiple regression model predicting SGHS scores included age and number of gambling activities (as covariates), impulsivity, social support, mainstream community connectedness, and erroneous gambling cognitions

^a^Variables not included in multiple regression analysis due to non-significant bivariate regression

^b^Number of gambling activities = total number of gambling activities participants had engaged with in past 12 months

^c^Variables not included in multiple regression analysis due to multicollinearity

^d^Variables not measured among the heterosexual participants

^e^Variables not included due to missing data

Bivariate regression analyses in the SMM group found that emotional informational support, positive social interaction and mainstream community connectedness significantly negatively predicted PGSI scores; and that number of gambling activities, gambling-related expectancies, illusion of control, predictive control, inability to stop gambling, interpretive bias, enjoyment/arousal expectancies, self-enhancement expectancies, over-involvement expectancies, impulsivity and perceived stigma significantly positively predicted PGSI scores. Emotional informational support, positive social interaction and mainstream community connectedness significantly negatively predicted SGHS scores; and age, number of gambling activities, gambling-related expectancies, illusion of control, predictive control, inability to stop gambling, interpretive bias, self-enhancement expectancies, over-involvement expectancies, emotional impact expectancies, impulsivity, psychological distress and perceived stigma significantly positively predicted SGHS scores (see Table 4).

The multiple regression analyses predicting PGSI scores for the heterosexual male and SMM groups are displayed in Table 4. For the heterosexual male group, the overall model for PGSI scores (after controlling for age, relationship status and number of gambling activities) significantly accounted for 57% of variance. The overall model for the SMM group (after controlling for number of gambling activities) significantly accounted for 50% of variance. In both groups, higher PGSI scores were significantly independently predicted by participating in a larger number of gambling activities, and higher levels of erroneous gambling cognitions and impulsivity.

The multiple regression analyses predicting SGHS scores for the heterosexual male and SMM groups are presented in Table 4. The overall model for the heterosexual male group (after controlling for number of gambling activities) accounted for 46% of variance in SGHS scores and the overall model for the SMM group (after controlling for age and number of gambling activities) accounted for 34% of variance. For both groups of participants, higher SGHS scores were significantly independently predicted by higher levels of erroneous gambling cognitions and impulsivity. A larger number of gambling activities was also a significant independent predictor for the SMM group.

Interaction terms were inspected for each of the variables which were included in the multiple regression analyses in Table 4 to examine whether each was a stronger predictor for PGSI scores and/or SGHS scores for SMM than heterosexual men. However, for both PGSI scores and SGHS scores, none of the interaction terms were statistically significant (see Appendix B in the supplementary materials).

## Discussion

This study was the first to examine both potential risk and protective factors for problem gambling severity and gambling-related harms among SMM. The inclusion of a comparison group of heterosexual men enabled an examination of the factors that may have been more pronounced among the SMM participants.

### Gambling behaviour, severity, and harms

Contrary to the first hypothesis, the heterosexual participants, on average, reported significantly higher levels of problem gambling severity compared with the SMM participants; but gambling-related harms did not differ between groups. These findings were unexpected, given that a previous study by Richard et al. [41] reported significantly higher levels of problem gambling among gay and bisexual male participants compared with heterosexual male participants. The finding may, however, be due to: (1) cultural differences as their study was conducted in the USA; or (2) the restriction of their sample to college student athletes, who have a greater risk for problem gambling compared with students who are not athletes [70].

The current study found a significantly greater proportion of heterosexual participants reported participating in any gambling activity, specifically casino table games, horse racing/greyhound race betting, sports betting, and keno compared with the SMM participants over the previous 12 months. Furthermore, on average, the heterosexual group had engaged in each of these four gambling activities significantly more frequently and spent significantly more money during each gambling session, than SMM participants. Once again, these findings contrast with those of Richard et al.’s [41] study, whereby significantly more gay male participants had participated in internet casino gambling and betting on horse racing/greyhounds than bisexual male and heterosexual male participants, and no significant differences in gambling frequency.

In order to interpret these unexpected findings, several differences in key demographic characteristics, potentially as a result of the convenience sampling employed in this study, between the two groups need to be considered. One potential explanation for the lower levels of problem gambling severity and gambling behaviour among SMM is that many do not have the same financial stability as heterosexual men, despite having achieved a higher level of education in Australia. This is supported by evidence that sexual and gender minority people are more likely to experience unequal employment opportunities and discriminatory attitudes in the workplace than heterosexual people [71–73]. Indeed, in the current study, a significantly greater proportion of SMM participants were unemployed and seeking work, while a significantly greater proportion of the heterosexual male participants were employed full-time. Sexual and gender minority people are also more likely to end up in lower paying jobs than they are qualified for as they find it difficult to secure employment with a non-discriminatory employer [74]. Therefore, the authors tentatively suggest that gambling frequently and/or with large amounts of money may not be a priority when there are other financial responsibilities to prioritise, such as housing and living expenses. While this may explain why the SMM group were less likely to report a high severity of problem gambling, it is inconsistent with the literature on gambling-related harms, which has found that lower socio-economic status is associated with experiencing more harms [75]. A second possible explanation, which is consistent with the gambling literature, is that the higher education levels of the SMM group in this study may have acted as a protective factor [33, 76, 77]. Further research is required to examine the impact of employment, socio-economic status and education on the gambling behaviour of sexual and gender minority men.

Despite these findings, SMM participants in this study were over-represented in all PGSI risk categories, displayed high rates of gambling-related harm, reported high rates of gambling participation, and reported high median gambling frequencies. They also reported high rates of participation on gambling activities that are associated with gambling problems and related harms, such as EGMs, horse/greyhound racing, and sports betting [78, 79]. Although caution is required in interpreting these findings due to the convenience- and over-sampling employed in this study, they suggest that further research using population-representative studies comparing gambling behaviour in SMM and heterosexual males is warranted.

### Potential risk factors for problem gambling severity and harms

Findings for the second hypothesis were mixed. Consistent with the broader gambling literature, higher levels of erroneous gambling cognitions were found to consistently significantly independently predict problem gambling severity and gambling-related harms for both SMM and heterosexual male participants [11, 12, 17, 80–82]. Higher levels of impulsivity were also found to be a significant predictor of both gambling outcomes which was consistent with the broader gambling literature [15, 19] and gambling research involving SMM [42, 83].

In contrast, hazardous alcohol use was associated with problem gambling severity, but not gambling-related harms, for heterosexual male participants but not SMM participants in the bivariate analyses, suggesting that this is a risk factor unique to heterosexual male participants. While this is consistent with other gambling research [9, 23, 81, 82], hazardous alcohol use was not an independent predictor in the multivariate analyses for heterosexual men, suggesting that other factors, such as erroneous gambling cognitions and impulsivity, better explain the variance in problem gambling severity for heterosexual males.

Psychological distress and gambling outcome expectancies were not included in the models predicting problem gambling severity and gambling-related harms due to multicollinearity issues. Bivariate analyses revealed that among the SMM group, psychological distress significantly positively predicted problem gambling severity but not gambling-related harms which was partially consistent with the broader gambling literature [84]. Nonetheless, these were new findings for gambling research involving SMM as a significant relationship between problem gambling severity and psychological distress has not been found elsewhere [83]; and although not significant, the relationship between psychological distress and gambling-related harm among SMM has not been investigated elsewhere. Furthermore, the findings that the gambling outcome expectancy scales of over-involvement expectancies, emotional impact expectancies, enjoyment/arousal expectancies, and self-enhancement expectancies significantly positively predicted problem gambling severity and/or related harms was also consistent with other gambling research, however, the finding that money expectancies were negatively, or not significantly, associated with problem gambling severity/related harms contrasts with previous research [13, 14, 17, 85]. Among the SMM group, these non-significant findings about money expectancies may be due to the traditionally masculine and heteronormative nature of gambling in Australia and the spaces that gambling is more likely to occur. In Australia, gambling spaces and advertisements tend to focus on heterosexual and traditionally masculine Australian identities, values, beliefs and practices (for an overview of the history of masculinity in Australia, see [86]), which may isolate most SMM or make gambling venues feel inaccessible [87]. This is especially true of sports betting which is described as a boisterous and masculine leisure activity [88], and is advertised in Australia using heteronormative tropes such as sexualised and objectified imagery of women, mateship, power, control, and social superiority [87]. Furthermore, many of the gambling spaces in Australia are based in pub locales which may be perceived as unsafe for SMM, who are more likely to frequent social spaces that are known to be LGBTIQ + friendly [89], but do not provide opportunities for unplanned gambling due to an absence of electronic gaming machines. Therefore, gambling among SMM may typically be a solitary activity rather than a social activity. This may reduce their likelihood of developing an inflated sense of gambling-related winnings among their peers, and thus lower their expectation to win money or make a profit from gambling.

None of the interactions between the potential risk factors and problem gambling severity and harms were significant, suggesting that none of these risk factors were more pronounced among SMM compared with heterosexual men. While further research may be needed to examine these specific risk factors, these findings can be used as a comparative benchmark for future research.

Two factors related to minority stress, perceived sexual and gender identity-related stigma and discrimination, were examined as potential risk factors for SMM. This was the first study to examine the potential influence of minority stress on problem gambling severity and gambling-related harms. While discrimination was not significantly associated with either of the two gambling outcomes, significant positive bivariate regressions between perceived stigma and problem gambling severity and gambling-related harms suggests SMM who experience more perceived sexual and gender identity-related stigma also gamble at more problematic levels and experience more gambling-related harms.

### Potential protective factors for problem gambling severity and harms

None of the potential protective factors were found to significantly predict problem gambling severity or gambling-related harms in the multiple regression models, thus not supporting the fourth hypothesis. Bivariate analyses, however, found higher levels of mainstream community connectedness significantly predicted lower levels of both gambling outcomes for SMM, but not heterosexual males, suggesting that this is may be a protective factor unique to SMM. Moreover, bivariate analyses revealed higher levels of all social support significantly predicted lower levels of problem gambling severity and harms for heterosexual males, which is consistent with the available gambling literature [90, 91]. These analyses, however, revealed that only emotional informational support and positive social interaction, and not tangible or affective support, were associated with both problem gambling severity and harms for SMM participants, suggesting that these forms of support are more protective for this minority subgroup. Consistent with previous research [92], resilience was significantly associated both gambling outcomes for the heterosexual male participants, but for neither gambling outcomes for SMM participants. Moreover, the SMM group reported significantly lower levels of resilience than the heterosexual group and lower levels of resilience than SMM in another Australian study involving SMM [93]. It is therefore possible that the SMM participants in the current study had particularly low levels of resilience which may have had an impact on the relationship with the gambling outcome measures. However, it is also possible that resilience may not be a protective factor against problem gambling for SMM. Despite these findings, none of the interactions between the potential protective factors and problem gambling severity and harms were significant, suggesting that none of these protective factors were more pronounced among SMM compared with heterosexual men. It is evident that further research examining the protective role of different facets of mainstream community connectedness, social support, and resilience in SMM, as well as the role of other potential protective factors, is required.

### The influence of male gender and sexual identity on problem gambling severity and harms

The outcomes from this study add new evidence to the gambling literature on risk and protective factors associated with problem gambling severity and gambling-related harms among SMM. Interestingly, in the multiple regression models, the same risk factors (erroneous gambling cognitions and impulsivity) were found to predict problem gambling severity and gambling-related harms in both participant groups. This suggests that the influence of male gender on gambling may be more influential than sexual identity. This idea is supported by research examining the influence of masculinity norms and values. As discussed, research has consistently found male gender to be associated with problem gambling (see review [15]) and indeed, SMM’s experiences include both identifying with a minority sexual identity and identifying as male [94, 95]. Traditional male gender role socialisation (also known as traditional masculinity) directs men away from self-care and instead encourages risky behaviours [96, 97], such as engaging in problem gambling to escape emotional pain [98, 99]. This is not exclusive to heterosexual men, however, as research has found that gay men who identify with traditional masculinity are more likely to engage in health risk behaviours, such as hazardous alcohol use and sexual risk behaviour [94]. However, considering the association between masculinity and heterosexuality [100], the additional minority-related stressors that SMM experience [37], and the lower levels of resilience that have been reported by SMM both in the current study and in previous research [93], future research should consider examining the intersection between masculinity, minority stress, and problem gambling among SMM.

### Study implications

An implication from this study relates to the need for community-level education initiatives. Many of the SMM in this study were engaged in higher-risk gambling activities, two-thirds were classified within the problem gambling and moderate-risk gambling PGSI categories, and 71% reported at least one gambling-related harm. It is therefore possible that there may be a low awareness about the harms related to gambling as some forms, such as EGMs, are perceived as entertainment and more socially acceptable [79, 101, 102]. Therefore, health promotion campaigns should be developed to educate SMM about the harms associated with gambling. Importantly, although problem gambling and gambling-related harms are overlapping but not synonymous constructs [51, 103, 104], the high rates of gambling-related harms reported by SMM in this study suggests that the campaign messages could shift the focus from problem gambling to gambling-related harms to avoid further stigmatising an already stigmatised and marginalised population.

### Study limitations

Consideration of some of the study limitations is warranted. First, while the large sample size, with an over-representation of SMM and people with gambling problems was a notable strength of this study, especially considering that minority populations are typically hard to reach [105, 106], caution is required in generalising the gambling estimates to the general population, given the convenience and over-sampled nature of the sample. Further research using population-representative studies comparing gambling behaviour in SMM and heterosexual males is required. Second, the survey was self-report and hence may be sensitive to under-reporting although studies have shown that gambling self-report data is reasonably reliable [107, 108]. Third, the terms ‘risk factor’ and ‘protective factor’ were used to refer to variables which may be associated with an increased risk and decreased risk for developing problem gambling/related harms, respectively. However, risk and protective factors are generally defined as antecedent variables or conditions that can predict the subsequent development of mental health disorders [109]. While cross-sectional studies provide some insight into the factors associated with problem gambling, they are unable to establish any level of causation. Thus, future longitudinal research is required to definitively identify risk and protective factors in the SMM population. Finally, only three people with intersex variations volunteered to participate in this study. The study therefore does not currently account for experiences of people with intersex variations.

## Conclusions

In conclusion, the outcomes from this study add new evidence to the gambling literature on problem gambling severity and gambling-related harms among SMM which can be used as comparative benchmarks for future research. Heterosexual men were found to report a significantly higher severity of problem gambling than SMM, as well as higher gambling frequencies and expenditures. The two groups, however, did not differ in terms of gambling-related harms. Moreover, two-thirds of the current study’s SMM group were classified as problem or moderate-risk gamblers. The same risk factors (erroneous gambling cognitions and impulsivity) were found to predict problem gambling severity and gambling-related harms in both participant groups. Future research examining the intersection between masculinity, minority stress, and problem gambling among SMM appears warranted.

## Supplementary Information

**Additional file 1**. **Appendix A:** Statistical group comparisons inpotential risk and protective factors. **Appendix B:** Moderation analyses.

## Data Availability

Data is available upon request. The survey instrument is accessible at OSF: https://mfr.osf.io/render?url=https%3A%2F%2Fosf.io%2Fd5mk6%2Fdownload.

## Abbreviations

AUDIT-C: The Alcohol use disorders identification test—consumption
BRS: Brief resilience scale
GEQ: Gambling expectancy questionnaire
GRCS: Gambling related cognition scale
K6: 6-Item Kessler psychological distress scale
LGBTIQ +: Lesbian, gay, bisexual, trans, intersex, queer, and other sexually and gender diverse
MOS-SS: Medical outcomes study social support survey
PGSI: Problem Gambling severity index
SGHS: Short gambling harms screen
SMM: Sexual minority men (i.e. bisexual, gay, pansexual, and other sexual orientations in which men have sex with men)
UPPS-P: The (Negative) Urgency subscale of the UPPS-P Impulsive Behaviour Scale
