# Supplementary material for: Risk and protective factors for the development of gambling-related harms and problems among Australian sexual minority men
Source: BMC Psychol. 2021 Jun 29;9:102. doi: 10.1186/s40359-021-00597-4 (PMC8240316; doi:10.1186/s40359-021-00597-4)
Supplement: Supplementary file 1 — Additional file 1. Appendix A: Statistical group comparisons inpotential risk and protective factors. Appendix B: Moderation analyses. [file 40359_2021_597_MOESM1_ESM.docx]

# Appendix A: Statistical Group Comparisons in Potential Risk and Protective Factors

Table A.1

*Comparisons in Potential Risk and Protective Factors Between Heterosexual Male and Sexual Minority Male Participants*

| Characteristics | Het. men | SMM | Inferential statistics | Effect size |
| --- | --- | --- | --- | --- |
| **Gambling-related expectancies^a^, *M* (*SD*)** | **14.2 (4.7)*** | **12.7 (5.4)** | ***Welch* (177.24) = 2.27, *p* = .025** | ***d* =-.30** |
| Illusion of control^a^, *M* (*SD*) | 8.3 (4.9) | 7.4 (4.6) | *t* (306) = 1.40, *p* = .162 | *d* = -.18 |
| Predictive control^a^, *M* (*SD*) | 17.2 (7.4) | 16.2 (7.3) | *t* (304) = 1.13, *p* = .261 | *d* = -.14 |
| Inability to stop gambling^a^, *M* (*SD*) | 13.4 (8.6) | 11.6 (7.3) | *Welch* (233.58) = 1.83, *p* = .069 | *d* = -.22 |
| **Interpretive bias^a^, *M* (*SD*)** | **14.1 (6.0)*** | **12.0 (5.8)** | ***t* (305) = 2.90, *p* = .004** | ***d* = -.35** |
| **Enjoyment/arousal expectancies^b^, *M* (*SD*)** | **40.9 (6.7)*** | **38.4 (8.1)** | ***t* (302) = 2.87, *p* = .004** | ***d* = -.35** |
| **Self-enhancement expectancies^b^, *M* (*SD*)** | **14.0 (5.0)*** | **11.8 (5.2)** | ***t* (304) = 3.56, *p*** *<* **.001** | ***d* = -.43** |
| Money expectancies^b^, *M* (*SD*) | 11.1 (3.9) | 10.3 (3.9) | *t* (305) = 1.83, *p* = .068 | *d* = -.21 |
| **Over-involvement expectancies^b^, *M* (*SD*)** | **14.5 (7.7)*** | **12.6 (7.0)** | ***t* (303) = 2.05, *p* = .041** | ***d* = -.25** |
| Emotional impact expectancies^b^, *M* (*SD*) | 9.7 (5.5) | 9.3 (5.2) | *t* (305) = .52, *p* = .601 | *d* = -.07 |
| Hazardous alcohol use^c^, *M* (*SD*) | 6.4 (2.8) | 5.9 (3.0) | *t* (288) = 1.25, *p* = .213 | *d* = -.17 |
| Impulsivity^d^, *M* (*SD*) | 2.3 (.8) | 2.5 (.7) | *t* (304) = -1.94, *p* = .054 | *d* = .26 |
| **Psychological distress^e^, *M* (*SD*)** | **14.1 (5.8)** | **15.8 (6.0)*** | ***t* (301) = -2.43, *p* = .016** | ***d* = .29** |
| **Resilience^f^, *M* (*SD*)** | **3.4 (.9)*** | **3.1 (.8)** | ***t* (304) = 3.45, *p* = .001** | ***d* = -.35** |
| **Emotional/informational support^g^, *M* (*SD*)** | **3.7 (1.1)*** | **3.4 (1.1)** | ***t* (298) = 2.42, *p* = .016** | ***d* = -.27** |
| **Tangible support^g^, *M* (*SD*)** | **3.8 (1.2)*** | **3.3 (1.2)** | ***t* (304) = 3.58, *p* < .001** | ***d* = -.42** |
| **Affectionate support^g^, *M* (*SD*)** | **4.0 (1.2)*** | **3.5 (1.3)** | ***t* (301) = 2.78, *p* = .006** | ***d* = -.41** |
| **Positive social interaction^g^, *M* (*SD*)** | **4.0 (1.1)*** | **3.6 (1.1)** | ***t* (303) = 2.83, *p* = .005** | ***d* = -.36** |
| Mainstream community connectedness^h^, *M* (*SD*) | 18.7 (4.6) | 17.7 (4.3) | *t* (303) = 1.80, *p* = .073 | *d* = -.22 |
| LGBTIQ+ community connectedness^h^, *M* (*SD*) | - | 16.9 (5.1) |  |  |
| Perceived stigma^i^, *M* (*SD*) | - | 16.3 (5.7) |  |  |
| Perceived discrimination, *M* (*SD*) |  |  |  |  |
| Not at all | - | 31 (31.0) |  |  |
| Not really | - | 21 (21.0) |  |  |
| Undecided | - | 9 (9.0) |  |  |
| Somewhat | - | 27 (27.0) |  |  |
| Very much | - | 12 (12.0) |  |  |

Note: Rows in bold indicate significant differences between groups. *d* = Cohen’s *d.* Φ = Phi. SMM = sexual minority men.

*Indicates that the proportion of respondents in that category from that group (either heterosexual male or sexual minority male participants) is significantly higher than the proportion of respondents from the other group.

^a^Gambling Related Cognition Scale (GRCS). Total score range = 23-161; Gambling-related expectancies score range = 4-28; Illusion of control score range = 4-28; Predictive control score range = 6-42; Inability to stop gambling score range = 5-35; Interpretive bias score range = 4-28.^b^Gambling Expectancy Questionnaire (GEQ). Enjoyment/Arousal score range = 8-56; Self-enhancement score range = 4-28; Money score range = 3-21; Over-involvement score range = 5-35; Emotional impact score range = 3-21. ^c^Alcohol Use Disorders Identification Test – Consumption (AUDIT-C). Score range = 0-12. ^d^The (Negative) Urgency subscale of the UPPS-P Impulsive Behaviour Scale. Score range = 1-4. ^e^6-item Kessler Psychological Distress Scale (K6). Score range = 0-24. ^f^The Brief Resilience Scale (BRS). Score range = 1-6.^g^The Medical Outcomes Study Social Support Survey (MOS-SS). Score range for the total and subscales = 1-5. ^h^Connectedness to the LGBT Community Scale. Score range = 7-28. ^i^Perceptions of Local Stigma Scale (PLS). Score range = 7-35.

# Appendix B: Moderation Analyses

Table B.1

*Multivariate Regressions Predicting PGSI Score and SGHS Score with Alcohol Use and Minority Sexual Identity*

|  | **PGSI** | | | **SGHS** | | |
| --- | --- | --- | --- | --- | --- | --- |
|  | ***B* (95% CI)** | ***t*** | ***p*** | ***B* (95% CI)** | ***t*** | ***p*** |
| (Constant) | **1.22 (.85, 1.60)** | **6.37** | **< .001** | **1.77 (.51, 3.03)** | **2.76** | **.006** |
| Age (in years) | -.01 (-.02, .00) | -1.18 | .237 | .01 (-.03, .04) | 0.29 | .773 |
| Relationship (ref=single) | -.10 (-.32, .13) | -.86 | .390 | -.04 (-.79, .71) | -0.09 | .925 |
| Gambling participation^a^ | **.17 (.12, .21)** | **6.56** | **< .001** | **.39 (.23, .56)** | **4.70** | **< .001** |
| Minority sexual identity | -.06 (-.30, .18) | -.47 | .641 | .11 (-.68, .91) | 0.28 | .783 |
| Alcohol use (centred score) | .04 (-.01, .09) | 1.55 | .122 | **.18 (.02, .35)** | **2.17** | **.030** |
| Alcohol use x minority sexual identity interaction | -.01 (-.09, .07) | -.26 | .794 | -.03 (-.30, .24) | -0.22 | .827 |
| *R*^2^ | .19 | | | .12 | | |
| *F* | 10.98*** | | | 6.51*** | | |

Note: Rows in bold indicate significant predictors. *B* = Unstandardised beta. CI = confidence interval. ****p* < .001.

^a^Gambling participation=total number of gambling activities participants had engaged with in past 12 months.

Table B.2

*Multivariate Regressions Predicting PGSI Score and SGHS Score with Impulsivity and Minority Sexual Identity*

|  | **PGSI** | | | **SGHS** | | |
| --- | --- | --- | --- | --- | --- | --- |
|  | ***B* (95% CI)** | ***t*** | ***p*** | ***B* (95% CI)** | ***t*** | ***p*** |
| (Constant) | **1.27 (.94, 1.59)** | **7.65** | **< .001** | **1.98 (.89, 3.07)** | **3.56** | **< .001** |
| Age (in years) | .00 (-.01, .01) | -.56 | .575 | .01 (-.02, .04) | 0.93 | .353 |
| Relationship (ref=single) | -.06 (-.25, .14) | -.59 | .552 | .02 (-.63, .68) | 0.06 | .949 |
| Gambling participation^a^ | **.14 (.10, .18)** | **6.74** | **< .001** | **.34 (.20, .48)** | **4.85** | **< .001** |
| Minority sexual identity | -.19 (-.40, .02) | -1.75 | .080 | -.36 (-1.06, .35) | -0.99 | .322 |
| Impulsivity (centred score) | **.64 (.49, .79)** | **8.33** | **< .001** | **2.10 (1.59, 2.61)** | **8.14** | **< .001** |
| Impulsivity x minority sexual identity interaction | -.11 (-.39, .17) | -.78 | .438 | -.61 (-1.54, .32) | -1.28 | .201 |
| *R*^2^ | .36 | | | .29 | | |
| *F* | 28.52*** | | | 20.50*** | | |

Note: Rows in bold indicate significant predictors. *B* = Unstandardised beta. CI = confidence interval. ****p* < .001.

^a^Gambling participation=total number of gambling activities participants had engaged with in past 12 months.

Table B.3

*Multivariate Regressions Predicting PGSI Score and SGHS Score with Erroneous Gambling Cognitions and Minority Sexual Identity*

|  | **PGSI** | | | **SGHS** | | | |  |
| --- | --- | --- | --- | --- | --- | --- | --- | --- |
|  | ***B* (95% CI)** | ***t*** | ***p*** | | ***B* (95% CI)** | ***t*** | ***p*** | |
| (Constant) | **1.37 (1.07, 1.66)** | **9.11** | **< .001** | | **2.20 (1.15, 3.26)** | **4.10** | **< .001** | |
| Age (in years) | .00 (-.01, .00) | -.88 | .381 | | .01 (-.02, .04) | .74 | .462 | |
| Relationship (ref=single) | .09 (-.09, .26) | .94 | .347 | | .42 (-.22, 1.05) | 1.29 | .196 | |
| Gambling participation^a^ | **.09 (.05, .12)** | **4.28** | **< .001** | | **.19 (.06, .33)** | **2.75** | **.006** | |
| Minority sexual identity | .05 (-.14, .23) | .50 | .620 | | .31 (-.36, .98) | .92 | .358 | |
| Erroneous gambling cognitions (centred score) | **.03 (.02, .03)** | **11.89** | **< .001** | | **.08 (.06, .10)** | **9.81** | **< .001** | |
| Erroneous gambling cognitions x minority sexual identity interaction | -.01 (-.01, .00) | -1.33 | .183 | | -.02 (-.05, .00) | -1.80 | .072 | |
| *R*^2^ | .48 | | | .35 | | | |  |
| *F* | 46.89*** | | | 26.97*** | | | |  |

Note: Rows in bold indicate significant predictors. *B* = Unstandardised beta. CI = confidence interval*.* ****p* < .001.

^a^Gambling participation=total number of gambling activities participants had engaged with in past 12 months.

Table B.4

*Multivariate Regressions Predicting PGSI Score and SGHS Score with Resilience and Minority Sexual Identity*

|  | **PGSI** | | | **SGHS** | | |
| --- | --- | --- | --- | --- | --- | --- |
|  | ***B* (95% CI)** | ***t*** | ***p*** | ***B* (95% CI)** | ***t*** | ***p*** |
| (Constant) | **1.20 (.84, 1.56)** | **6.54** | **< .001** | **1.81 (.61, 3.00)** | **2.97** | **.003** |
| Age (in years) | .00 (-.01, .01) | -.79 | .432 | .01 (-.02, .04) | 0.56 | .574 |
| Relationship (ref=single) | -.09 (-.31, .12) | -.84 | .403 | -.10 (-.81, .62) | -0.27 | .787 |
| Gambling participation^a^ | **.17 (.12, .21)** | **7.11** | **< .001** | **.42 (.26, .57)** | **5.39** | **< .001** |
| Minority sexual identity | -.13 (-.36, .11) | -1.04 | .297 | -.20 (-.98, .58) | -0.51 | .614 |
| Resilience (centred score) | **-.27 (-.42, -.12)** | **-3.48** | **.001** | **-.94 (-1.44, -.44)** | **-3.68** | **< .001** |
| Resilience x minority sexual identity interaction | .08 (-.18, .35) | .60 | .546 | .31 (-.56, 1.19) | 0.70 | .483 |
| *R*^2^ | .21 | | | .15 | | |
| *F* | 13.39*** | | | 8.61*** | | |

Note: Rows in bold indicate significant predictors. *B* = Unstandardised beta. CI = confidence interval*.* ****p* < .001.

^a^Gambling participation=total number of gambling activities participants had engaged with in past 12 months.

Table B.5

*Multivariate Regressions Predicting PGSI Score and SGHS Score with Social Support and Minority Sexual Identity*

|  | **PGSI** | | | **SGHS** | | |
| --- | --- | --- | --- | --- | --- | --- |
|  | ***B* (95% CI)** | ***t*** | ***p*** | ***B* (95% CI)** | ***t*** | ***p*** |
| (Constant) | **1.25 (.90, 1.61)** | **6.84** | **< .001** | **1.88 (.67, 3.09)** | **3.05** | **.002** |
| Age (in years) | -.01 (-.02, .00) | -1.85 | .064 | -.01 (-.04, .03) | -.30 | .768 |
| Relationship (ref=single) | .02 (-.20, .24) | .15 | .878 | .18 (-.56, .92) | .48 | .631 |
| Gambling participation^a^ | **.17 (.13, .22)** | **7.50** | **< .001** | **.44 (.29, .59)** | **5.72** | **< .001** |
| Minority sexual identity | -.12 (-.35, .11) | -.99 | .322 | -.16 (-.93, .62) | -.39 | .694 |
| Social support (centred score) | **-.25 (-.38, -.12)** | **-3.83** | **< .001** | **-.59 (-1.02, -.16)** | **-2.67** | **.007** |
| Social support x minority sexual identity interaction | .02 (-.19, .23) | .20 | .839 | -.21 (-.92, .50) | -.57 | .566 |
| *R*^2^ | .23 | | | .14 | | |
| *F* | 14.59*** | | | 8.21*** | | |

Note: Rows in bold indicate significant predictors. *B* = Unstandardised beta. CI = confidence interval*.*  ****p* < .001.

^a^Gambling participation=total number of gambling activities participants had engaged with in past 12 months.

Table B.6

*Multivariate Regressions Predicting PGSI Score and SGHS Score with Community Connectedness and Minority Sexual Identity*

|  | **PGSI** | | | **SGHS** | | | |  |
| --- | --- | --- | --- | --- | --- | --- | --- | --- |
|  | ***B* (95% CI)** | ***t*** | ***p*** | | ***B* (95% CI)** | ***t*** | ***p*** | |
| (Constant) | **1.18 (.81, 1.55)** | **6.30** | **< .001** | | **1.72 (.50, 2.93)** | **2.76** | **.006** | |
| Age (in years) | .00 (-.01, .01) | -.90 | .370 | | .01 (-.03, .04) | .46 | .643 | |
| Relationship (ref=single) | -.12 (-.34, .10) | -1.04 | .297 | | -.20 (-.93, .53) | -.54 | .591 | |
| Gambling participation^a^ | **.17 (.12, .22)** | **7.25** | **< .001** | | **.43 (.28, .59)** | **5.55** | **< .001** | |
| Minority sexual identity | -.07 (-.31, .16) | -.62 | .538 | | -.04 (-.82, .74) | -.11 | .916 | |
| Community connectedness (centred score) | -.02 (-.04, .01) | -1.01 | .313 | | -.03 (-.12, .07) | -.56 | .576 | |
| Community connectedness x minority sexual identity interaction | -.02 (-.07, .03) | -.79 | .432 | | -.13 (-.30, .05) | -1.42 | .155 | |
| *R*^2^ | .18 | | | .11 | | | |  |
| *F* | 11.11*** | | | 6.48*** | | | |  |

Note: Rows in bold indicate significant predictors. *B* = Unstandardised beta. CI = confidence interval*.*  ****p* < .001.

^a^Gambling participation=total number of gambling activities participants had engaged with in past 12 months.
